# Rationale for investigating the use of anifrolumab in neuropsychiatric systemic lupus erythematosus: a combined narrative and case-based systematic literature review

**DOI:** 10.3389/fimmu.2026.1750071

**Published:** 2026-02-11

**Authors:** Elisabetta Chessa, Fabio Congiu, Giulia Rizzo, Marta Paola Pireddu, Elena Ragusa, Alberto Floris, Alberto Cauli, Matteo Piga

**Affiliations:** 1 Rheumatology Unit, Azienda Ospedaliera Universitaria (AOU) Cagliari, Cagliari, Italy; 2 Rheumatology, Department of Medical Sciences and Public Health, University of Cagliari, Cagliari, Italy

**Keywords:** alpha interferon, anifrolumab, neurological manifestations, systemic lupus erythematosus, therapeutic effect

## Abstract

**Background:**

Neuropsychiatric (NP) involvement represents one of the major challenges in Systemic Lupus Erythematosus (SLE), often requiring individualized therapeutic strategies. While anifrolumab inhibits the type I interferon receptor 1 (IFNAR1) and is approved for the treatment of moderate-to-severe SLE, randomized controlled trials have not evaluated its efficacy in NPSLE.

**Methods:**

We examined the pathophysiological rationale for inhibiting IFN-α using anifrolumab in NPSLE. To supplement this, we report an original case of NPSLE successfully treated with anifrolumab, along with similar cases identified through a systematic literature review (SLR) of Medline/PubMed and Embase, performed in accordance with PRISMA and CABARET guidelines.

**Results:**

Overexpression of IFN-α is linked to neurological symptoms in patients with inflammatory NPSLE, such as psychosis and seizures. Blocking the IFNAR1 with anifrolumab provides a direct rationale for treating this subset of NPSLE. The SLR identified seven case reports of female patients with inflammatory NPSLE where anifrolumab was used as a rescue therapy following conventional treatment failure. NPSLE manifestations were heterogeneous, including psychosis, headache, and acute confusional state, which limits the generalizability of our findings. A 52-year-old female with SLE and seizures from our Lupus Clinic who received anifrolumab after failing multiple treatments was also reported. After an average of 11.7 months, all patients showed improvement, 87% (7 out 8) achieving complete NP symptom resolution and 62% reaching SLE remission. No emerging safety issues were reported.

**Conclusion:**

Preliminary observations suggest a potential benefit of anifrolumab in NPSLE, but evidence remains insufficient to establish clinical efficacy and warrants further controlled studies.

## Introduction

Systemic lupus erythematosus (SLE) is a complex, multisystem chronic autoimmune disease. Neuropsychiatric (NP) involvement is one of the most complex and challenging features of SLE and may affect the central, peripheral, and autonomic nervous systems. NPSLE can vary from mild to severe, be focal or diffuse, and present as acute or chronic (1). By negatively impacting the patient’s quality of life and increasing the mortality rate by up to ten times, NPSLE management represents an unmet need for patients with SLE (2–5).

Two primary, complementary, pathogenetic pathways underlying NPSLE have been identified (6–8). An ischemic pathway involving large and small blood vessels, mainly mediated by antiphospholipid (aPL) antibodies, immune complexes with complement activation, and intravascular thrombosis. The second is an autoimmune-mediated neuroinflammatory pathway characterized by increased blood–brain barrier permeability, intrathecal migration of brain-reactive autoantibodies, local production of immune complexes and inflammatory mediators, and the release of pro-inflammatory cytokines, including type I IFNs, such as IFN-α. Therapeutic decisions are tailored to individual patients and are based on the suspected pathogenic mechanisms, the attribution, and severity of NPSLE manifestations (9). EULAR recommends antiplatelet/anticoagulants for atherothrombotic/aPL events, as well as glucocorticoids and immunosuppressants for active inflammatory NPSLE (10). Evidence for the biologic drugs anifrolumab and belimumab in treating NPSLE is limited because active, severe cases were excluded from randomized controlled trials (RCTs), and belimumab is underused in real-world NPSLE treatment (10). However, anifrolumab has recently been approved in several countries for the treatment of moderate-to-severe SLE as an add-on therapy to standard treatment, and real-world reports on its potential use in patients with NPSLE are emerging.

To provide a rationale and clinical context for the use of anifrolumab in NPSLE, we present a narrative review of the potential mechanistic and a case-based systematic literature review (SLR) of real-world cases.

## Methods

We conducted a narrative review to critically analyze the pathogenic mechanisms underlying the potential role of anifrolumab in NPSLE.

Thereafter, Medline/PubMed and Embase databases were searched, in any language, for articles reporting patients with NPSLE treated with anifrolumab. The following search string was used: for Pubmed: ((“neuropsychiatric”[All Fields] OR “neurological manifestation”[All Fields] OR “psychiatric manifestation”[All Fields] OR “systemic lupus erythematosus”[All Fields] OR “epilepsy”[All Fields]) AND “anifrolumab”[All Fields]); for Embase: (‘systemic lupus erythematosus’/exp OR ‘systemic lupus erythematosus’:ti,ab OR sle:ti,ab) AND (‘neuropsychiatric lupus’/exp OR ‘neuropsychiatric systemic lupus erythematosus’:ti,ab OR npsle:ti,ab OR ‘epilepsy’/exp OR ‘psychosis’/exp) AND (‘anifrolumab’/exp OR anifrolumab:ti,ab).

**Figure 1:**
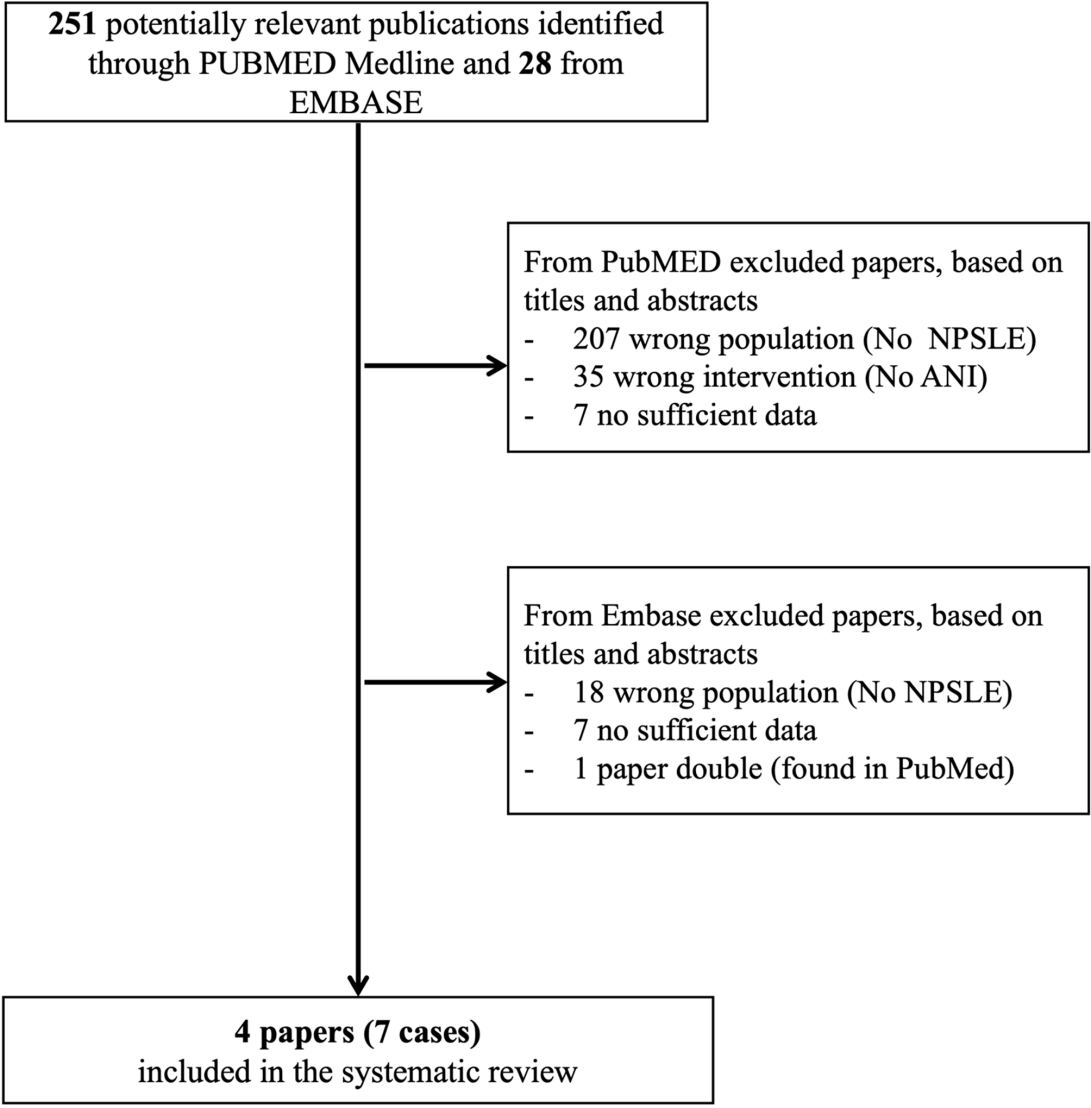
Flowchart of the SLR for articles reporting patients with NPSLE treated with anifrolumab.

Two authors (GR and EC) independently conducted the SLR from inception to 16th July 2025, initially based on titles and abstracts, and excluded studies that did not focus on the NPSLE population or did not mention the use of anifrolumab. Data from grey literature were excluded because there is no clear quality hierarchy for this data. Additional papers, including conference abstracts, were identified by reviewing the references of the selected studies. All cases of SLE patients with NP manifestations according to the 1999 ACR nomenclature (1) treated with anifrolumab were included in the study. Demographic data (age, sex), clinical data (disease duration, active clinical domains at anifrolumab initiation, and serology), previous and ongoing treatments, type of neuropsychiatric manifestations, laboratory and instrumental findings, follow-up, and outcomes were evaluated. To classify NPSLE manifestations as ischemic or inflammatory, we applied the algorithm proposed by Zirkzee et al. (11). In brief, ischemic NPSLE was defined by the presence of antiphospholipid antibodies or cardiovascular risk factors, along with infarction on brain magnetic resonance imaging (MRI) or thrombosis in MR angiography (MRA), consistent with NP symptoms. In case ischemic MRI or MRA lesions were absent or not explanatory for symptoms, inflammatory NPSLE was defined by the presence of at least one item of the following criteria: radiologic (e.g., white matter lesions on brain MRI), clinical (indication for immunosuppressive therapy for other active SLE manifestations, regardless of NPSLE), serological (complement consumption), anamnestic (e.g., resolved or reduced symptoms after immunosuppressive therapy). Neuropsychiatric outcomes were assessed clinically, according to the physician’s judgment, and categorized as complete resolution (disappearance of neuropsychiatric symptoms), improvement (incomplete or partial resolution), stability (no change in neuropsychiatric symptoms), or worsening (the development of more severe or new neuropsychiatric manifestations or death) between the time to starting Anifrolumab and the last visit (12). Studies lacking these data were excluded. Disagreements between investigators were solved by consensus. The research was conducted in accordance with the PRISMA checklist (13), and the results are presented in accordance with the Case-Based Review Standards (CABARET) (14). The PRISMA flow chart is presented in **Figure 1**. **A** detailed case of a patient with refractory manifestations, including NP involvement, who was successfully treated with anifrolumab, was retrieved from our Lupus Clinic and reported. Written informed consent was obtained from the patient. The Joanna Briggs Institute (JBI) critical appraisal tool was applied to the presented cases (15). PROSPERO registration was not completed. A search of the PROSPERO database for a review protocol examining the rationale, clinical efficacy, and safety for the specific use in NPSLE yielded no results (2 January 2026).

## Rationale for using anifrolumab in NPSLE

Several studies have analyzed potential pathogenetic pathways underlying NPSLE, focusing primarily on the roles of autoantibodies, blood-brain barrier dysfunction, and cytokines. The role of IFN-α has been increasingly investigated.

Type I interferons are overexpressed in 50-70% of SLE patients, with plasma levels 100-fold higher than those in healthy controls and less pronounced differences in cerebrospinal fluid (CSF) levels (16–18). However, the differences between SLE and NPSLE patients remain less conclusive (17, 18). Varley et al. did not find differences in IFN-α levels between SLE patients with and without NP involvement (17). On the other hand, studies that explored specific NP manifestations described significantly higher levels of IFN-α in patients with diffuse, inflammatory events, as cognitive impairment (19), acute confusional state (20), psychosis, and seizure (21). A case report of NPSLE with seizures showed that, even when IFN-α levels were not elevated in the CSF, post-mortem brain immunohistochemistry revealed IFN-α in neurons and microglia (21). Structural and functional brain MRI alterations due to elevated IFN-α levels have also been reported (19).

**Figure 2:**
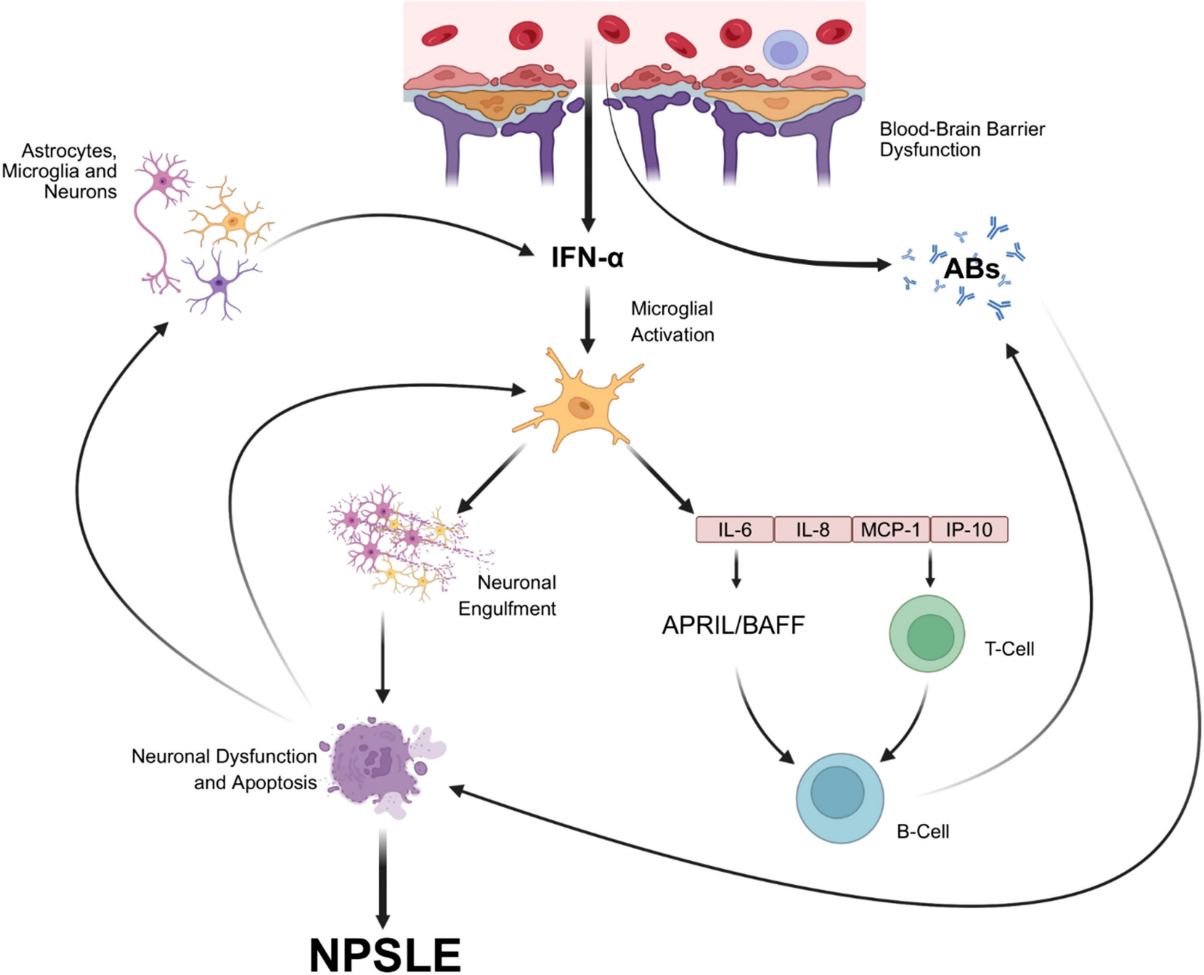
Cartoon showing pathogenetic mechanisms explaining the potential role of IFN-α in NPSLE development. Through disruption of the blood-brain barrier, especially when the disease is active (42), IFN-α can permeate and diffuse into the CNS (43). However, IFN-α may also be produced within the CNS by neurons, astrocytes, and microglia, which have been identified as the primary source of type-1 IFN in various neurological conditions, including viral infections, autoimmune disorders, and SLE (44–46). In particular, in the CNS, IFN-α may activate a distinct microglial subset that engulfs neurons by internalizing neuronal components or whole neurons, a process that results in neuronal damage and apoptosis (47). The interaction of IFN-α with microglia also triggers the production of inflammatory cytokines, such as IL-6, IL-8, monocyte chemoattractant protein-1 (MCP-1), and IFN-γ-induced protein-10 (IP-10), as well as cytotoxic lymphocytes and dendritic cells, all of which have been identified as associated with NPSLE pathogenesis (48–54).

The immunopathogenic and pro-inflammatory mechanisms explaining the potential role of IFN-α in NPSLE development are summarized in **Figure 2**. Persistent high serum IFN-α levels may also affect glutamate metabolism, which is implicated in behavioral mechanisms. An increase of glutamate in the basal ganglia, cingulate cortex (22), and in hippocampal neurons and the activation of the GluN2A subunit of the N-methyl-D-aspartate receptor (NR2) have been documented (23). This induces excitatory and inhibitory postsynaptic potentials, which are involved in epileptiform discharges associated with seizures (24) and in memory and cognitive alterations. A link is supported by numerous case reports that documented the onset of seizures following IFN-α therapy prescribed as antiviral therapy (25, 26). Studies conducted in murine models have revealed interesting implications of IFNs for neurological function. Intravenous injection of IFN in lupus-prone mice induced mood and cognitive alterations (27), and treatment with an anti-IFN-α/β receptor resulted in clinical and histological improvement (28). Huang et al. found that in MRL/lpr mice, an elevated peripheral signature was associated with anxiety and fatigue (29). Another recent study investigating the effect of IFNAR deletion showed that several NPSLE-associated phenotypes reversed and that microglial levels were reduced (30).

Anifrolumab, a fully human IgG1κ monoclonal antibody, targets and inhibits the type I interferon receptor IFNAR1, thereby blocking all type I IFN signaling. By preventing IFNAR1 engagement, anifrolumab disrupts receptor dimerization and activation, thereby inhibiting JAK-STAT signaling and the transcription of IFN-inducible genes (31, 32). Additionally, anifrolumab induces rapid internalization of IFNAR1 from the surface of immune cells, reducing the availability of the receptor for ligand binding and further attenuating the pathogenic IFN-driven response. A recent weighted gene co-expression network analysis revealed that the IFN gene module was notably upregulated in a subgroup of NPSLE patients, for whom in silico prediction algorithms indicated a higher likelihood of response to anifrolumab (33).

These findings support IFN-α as a potential driver of the subset of NPSLE manifestations driven by the inflammatory pathway, providing a rationale for inhibiting the type I interferon receptor IFNAR1 and blocking type I IFN signaling with anifrolumab.

## Systematic literature review

The SLR identified 279 potentially relevant publications, but only 4 were selected. A total of 7 relevant case reports of inflammatory NPSLE treated with anifrolumab were retrieved (34–37). Neuropsychiatric cases included two with acute confusional state (one with mood disorder), acute psychosis, headache, cerebral vasculitis, aseptic meningitis, and CIDP. All NP manifestations were attributed to SLE by expert opinion. The key clinical characteristics and outcomes are summarized in **Table 1**.

**Table 1:** Demographic, clinical, therapeutic, and outcome data of the cases retrieved from the SLR (1-7) and of the present case (8).

CASE	Sex (Age)	Disease duration (years)	Disease domains	AutoAb	NP manifestations	Failed prior therapy	Concomitant therapy to Iv ANI 300mg/m	CSF analysis	Ancillary investigations	Time to response (months)	Follow up (months)	Outcome
1 (34)	F (29)	NR	Cutaneous, Hematologic	NR	Acute psychosis	NR	GC iv	Negative	MRI (normal). Infections work-up: negative	6	6	NP Resolution; Remission (DORIS) without GCs
2 (35)	F (NR)	NR	Cutaneous, Hematologic Musculoskeletal	SSA APL-	Lupus headache	GC, HCQ, MTX, BEL	GC	Not performed	MRI (normal)	1	6	NP Improvement ; Remission (DORIS)
3 (36)	F (29)	0	Cutaneous, Constitutional	dsDNA, SSA, SSB, Sm, RNP, RibP APL-	Acute confusional state	GC iv, CYC	HCQ+ GC os	IL6 levels 17.4 pg/mL§	Serum IFN-α (1212 to 62 fg/mL)	1	22	NP resolution; Remission (DORIS)
4 (36)	F (21)	7	Vasculitic, Cutaneous	dsDNA, RNP APL-	Previous acute confusional state, Mood and anxiety disorders	GC iv, CYC, PEX, TAC, MMF, BEL	GC os	IL6 levels 17 pg/mL§	Mental status evaluation	14	14	NP resolution; Hospitalizations eliminated, psychotropic therapy halved
5 (36)	F (35)	18	Cutaneous	dsDNA, Sm, RNP, aCL, β2-GPI	Subarachnoid hemorrage	GC, TAC, MMF, AZA, BEL	AZA	Not performed	ce-MRI (microaneurysms in a branch of the basilar artery owing the possibility of a cerebral vasculitis), serum IFN-α (200 to 35 fg/ml)	12	16	NP resolution; no recurrence of SAH, Disappearance of microaneurysm
6 (36)	F (21)	7	Cutaneous, Musculoskeletal Serositic	RNP APL-	Aseptic meningitis	GC, TAC, BEL	any	Not performed	None	10	10	NP resolution; GC up to 2 mg/day with no aseptic meningitis flare
7 (37)	F (53)	26	Cutaneous, Hematologic, Musculoskeletal Vasculitic	dsDNA, SSA, SSB, APL-	Demyelinating polyneuropathy	GC iv, HCQ, AZA, RTX	MPRE 500 mg for 5 days + IVIg 100 g for 5 days and 50 g for 5 days	Not performed	Nerve conduction studies, muscle strenght scales (I-RODS 0 to 5 points; MRC 0 to 3 points; INCAT 10 to 8 points)	3	12	NP resolution; Remission (DORIS)
8 (present case)	F (53)	16	Cutaneous, Hematologic, Musculoskeletal Vasculitic	dsDNA, Sm, RNP, SSA, LAC, aCL, β2-GPI, RibP	Seizures	AZA + MMF + HCQ + BEL	PDN 10 mg/d, HCQ 400/d	Not performed	MRI (WMHI + left parietal area of resolved infarct), EEG (irritative distress projected to left hemispheric expression)	3	34	NP resolution; Remission (DORIS)

NR, not reported; AutoAb, Autoantibodies; APL_ antiphospholipids; anti-RibP, anti-ribosomal P protein; Iv, intravenous; GC, glucocorticoid; CYC, Cyclophosphamide; TAC, Tacrolimus; MMF, mycophenolate mofetil; BEL, Belimumab; AZA, Azathioprine; RTX, Rituximab; HCQ, Hydroxychloroquine; ANI, Anifrolumab; m, monthly; NP, Neuropsychiatric; IvIg, Intravenous Immunoglobulins; PEX, plasmapheresis. MRI Magnetic Resonance Imaging; CSF cerebrospinal fluid; I-RODS, Inflammatory Rasch-built Overall Disability Scale; MRC Medical Research Council; INCAT, Inflammatory Neuropathy Cause and Treatment; PDN, prednisone; § IL-6 reference levels:< 4.3 pg/mL.

**Figure 3:**
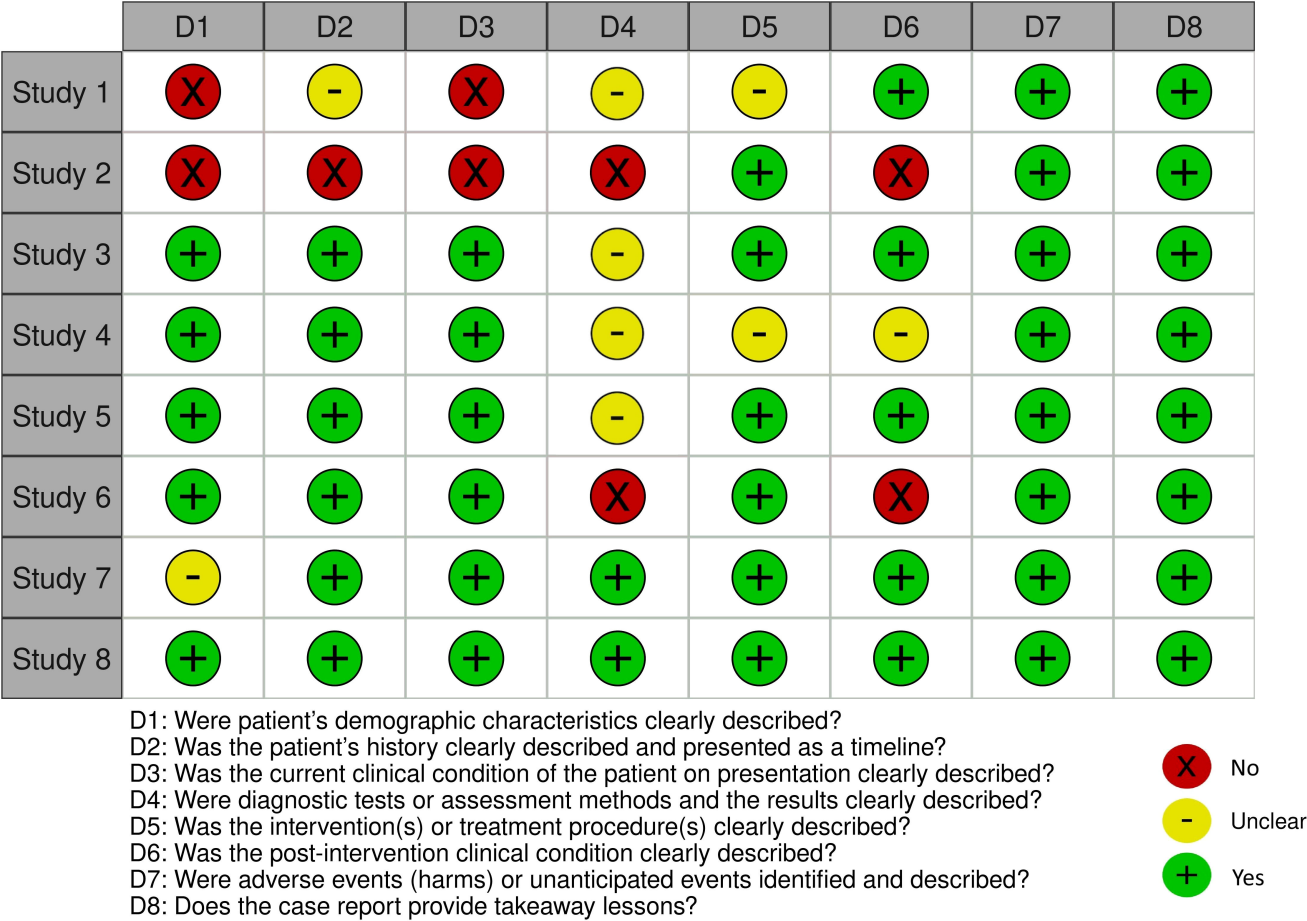
Traffic light plot displaying the quality of the case reports retrieved from the SLR (Cases 1-7) and of our case (Case 8) according to the Joanna Briggs Institute Critical Appraisal tool.

All patients were female, with a mean age of 31.3 (± 11.9) years and a mean disease duration of 11.6 (± 10.3) years. Anifrolumab 300 mg intravenous every 4 weeks was a second-line, or subsequent, treatment for inadequate disease activity control despite background immunosuppressive therapy. Clinically active involvement, other than NPSLE, included refractory cutaneous manifestations (100%), musculoskeletal (57%), hematologic (43%), vasculitic (29%), serositic (29%), and constitutional (14%) involvement. Following anifrolumab administration, with a minimum follow-up period of 6 months and a maximum of 22 months (mean time to outcome 11.7 ± 5.5 months), all patients showed improvement in neuropsychiatric symptoms, with complete resolution in 6 out of 7 patients (86%), improvement in 1 out of 7 (14%), and 4 out of 7 (57%) achieving SLE remission. No emerging safety issues were reported. The quality of the retrieved cases was good, except for cases 1 and 2, as assessed using the JBI critical appraisal tool (**Figure 3**).

## Case report

**Figure 4:**
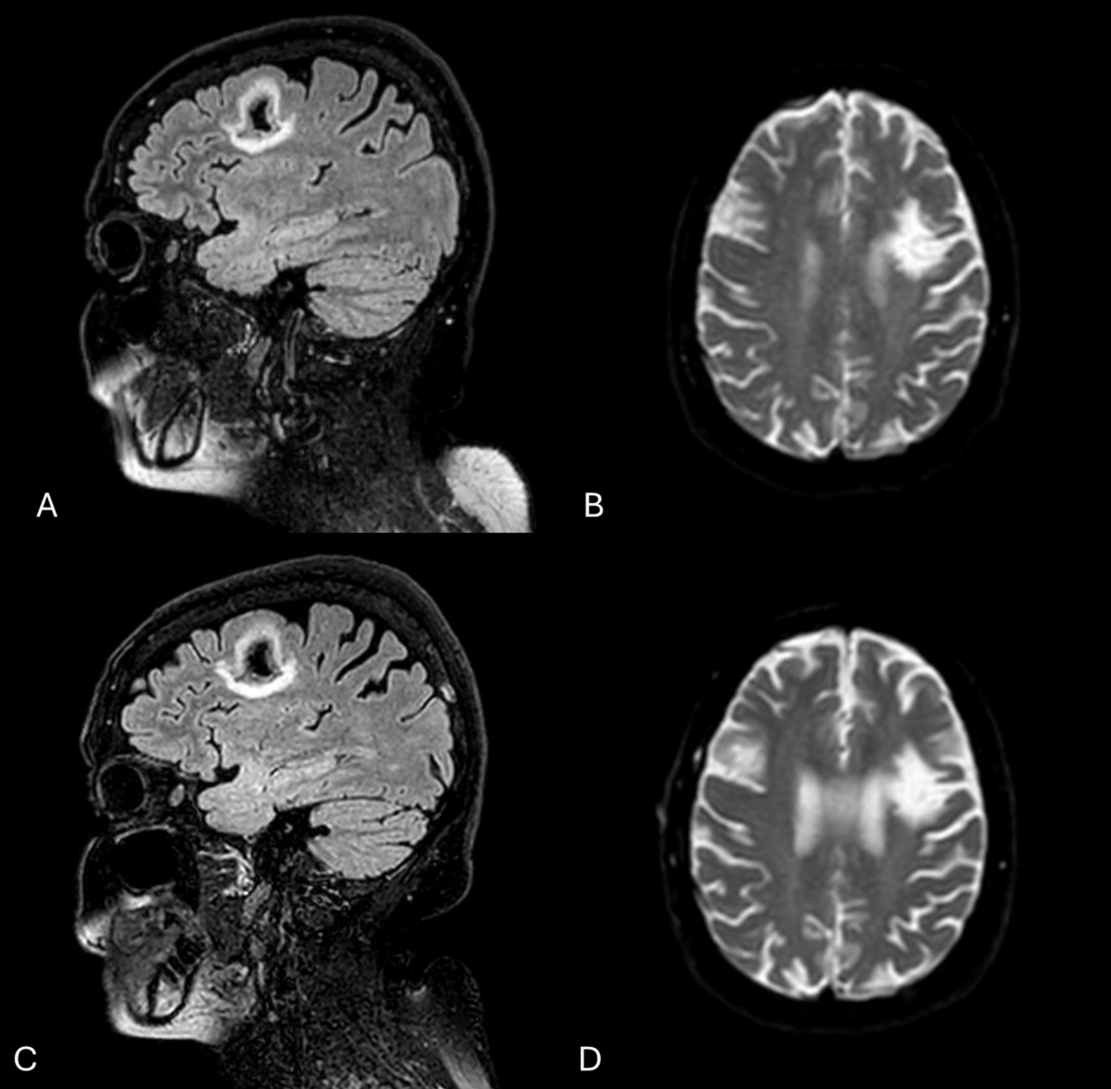
Brain MRI performed in 2014 showing a resolved left parietal infarct on FLAIR **(A)** and DWI **(B)** sequences. Brain MRI images, FLAIR **(C)** and DWI **(D)**, obtained in 2022, were unchanged.

A 52-year-old Caucasian woman was admitted to our unit in March 2014 due to a lupus flare with extensive cutaneous vasculitis, malar rash, thrombocytopenia, and leukopenia. One year before admission, she withdrew azathioprine for unspecified reasons. She had a 16-year history of SLE and antiphospholipid (aPL) syndrome (APS) diagnosed while presenting with polyarthritis, frontoparietal stroke, lymphopenia, antinuclear antibodies (ANA), anti-double-stranded (ds)DNA, anti-Ro/SSA, anti-Sm, anti-RNP, Lupus anticoagulant (LAC), anti-Cardiolipin (aCL) IgG, and low complement levels. She reported a history of partial seizures starting a year after the stroke, diagnosed as secondary to cerebral damage by a neurologist, and successfully treated with levetiracetam 250 mg twice daily. Given the lack of active SLE manifestations requiring immunosuppressive therapy, the explanatory MRI lesions, and the effectiveness of symptomatic treatment, this was classified as an ischemic NP manifestation. No NP events were recorded during the 6 months prior to and during admission. Brain conventional MRI showed deep ventricular white matter hyperintensities and a left parietal area of resolved infarct (**Figure 4**). The electroencephalogram showed signs of cerebral irritative distress projected to predominantly left hemispheric expression. She was dismissed with severe SLE and APS, but no active NPSLE, and treated with cyclophosphamide 500 mg every 2 weeks for 3 months (EuroLupus regimen), followed by mycophenolate mofetil (MMF) 2 grams/day, scheduled tapering of prednisone (PDN), and warfarin with an international normalised ratio (INR) target between 2 and 3. Hydroxychloroquine (HCQ) was added and then discontinued after only 3 months due to the development of tachyarrhythmia and INR dysregulation.

**Figure 5:**
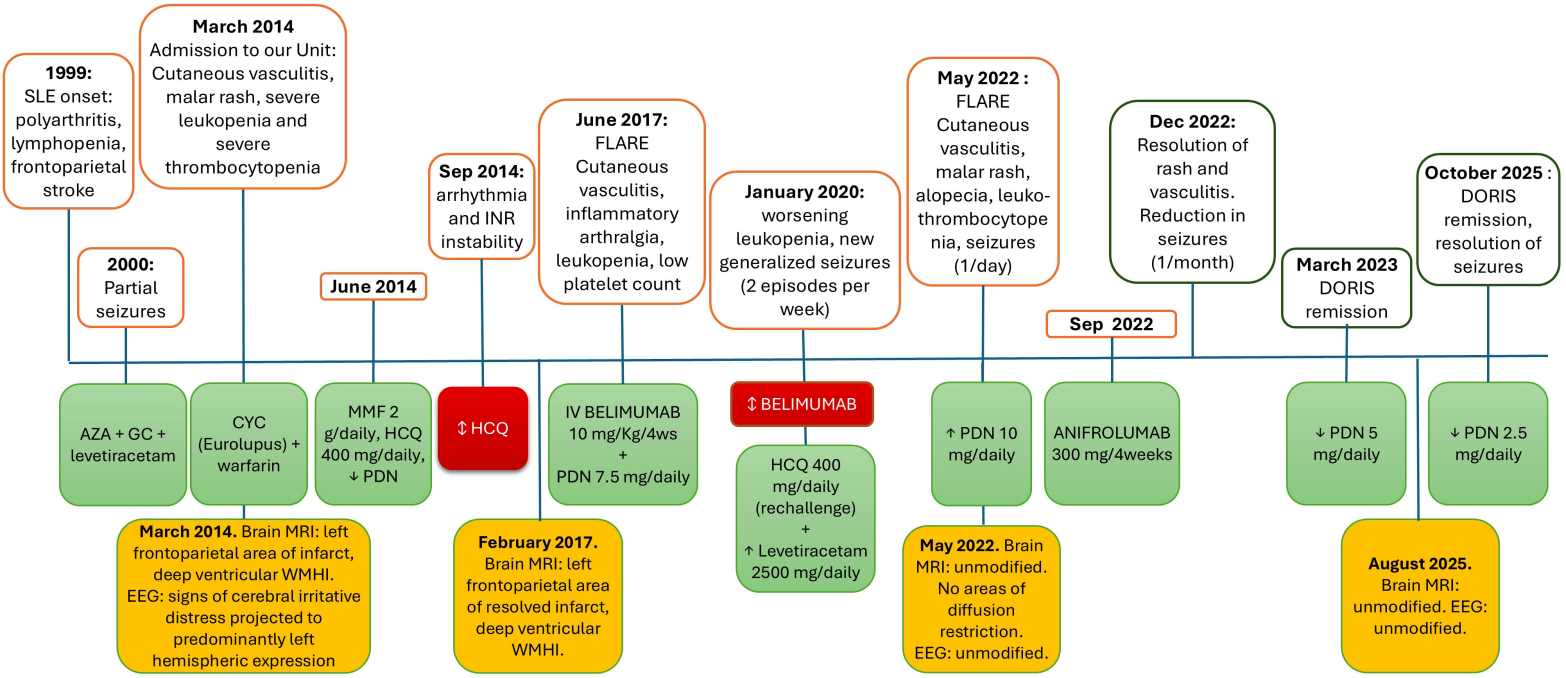
Timeline showing manifestations of the disease and corresponding treatments from diagnosis to the start of therapy. Legend: SLE, Systemic Lupus Eythematosus; AZA, azathioprine; GC, glucocorticoids; MRI, Magnetic resonance imaging; WMHI, white matter hyperintensities; EEG, electroencephalogram; CYC, cyclophosphamide; MMF, mofetil mycophenolate; HCQ, hydroxychloroquine; BLM, belimumab; PDN prednisone; ↓reduction; ↑increase; ↕ withdrawal.

In June 2017, she suffered from a flare with hand and foot vasculitis, inflammatory arthralgias, and thrombocytopenia. Belimumab (BLM) 10 mg/kg every 4 weeks was added to background treatment with MMF 2 g/day and PDN 7.5 mg/day. In January 2020, BLM was discontinued due to worsening leukopenia (<2000/μl). Over the years, partial seizures evolved into generalized and recurred twice a week, leading to an increase in levetiracetam dosage up to 2500 mg/day. A rechallenge with HCQ 5 mg/kg/day was performed without adverse events. In May 2022, after tapering the daily prednisone dose to 5 mg/day, she experienced a disease flare with recurrence of hand vasculitis, malar rash, alopecia, worsening thrombocytopenia (95000/μl), leukopenia (1710/μl), generalized seizures once a day and serological abnormalities (C3–60 mg/dl (nv <85 mg/dl), C4–24 mg/dl, anti-dsDNA 222 UI/ml (nv<20 UI/ml), anti-RibP 101 UI/ml (nv<18 UI/ml)) (CLASI-A=2, cSLEDAI-2K=22). Brain conventional MRI was unchanged. Given the generalized feature of seizures, the lack of new ischemic MRI lesions, the concurrent active clinical and serological manifestation, and the requirement of immunosuppressive treatment, this was classified as an inflammatory NP manifestation. In September 2022, she received her first monthly dose of anifrolumab 300 mg intravenously, along with an increase in PDN to 10 mg/day (**Figure 5**). At the 3-month follow-up visit, the rash had disappeared, the hand vasculitis had vanished, and she reported a lowering of epileptic crises to once a month. We pursued lowering glucocorticoids, and after 6 months of treatment, she was in clinical remission (cSLEDAI-2K=0, PDN 5mg/day). PDN was progressively lowered to 2.5 mg/day. At her last visit in May 2025, she was still in remission, without clinical activity (last epileptic crisis 6 months before, no cutaneous and vasculitic involvement, 4400/μl WBC, PLT 168000/μl), or serological activity (C3–86 mg/dl, C4–20 mg/dl, anti-dsDNA 16 UI/ml) (**Table 1**). The complete response of generalized seizures to immunosuppressive treatment further supports their inflammatory pathogenesis.

## Discussion and future perspectives

This study summarizes eight cases of NPSLE successfully treated with anifrolumab, seven identified through the SLR and one from our cohort. Although these findings remain anecdotal, the established involvement of type I IFN pathways in NPSLE pathogenesis provides a plausible rationale for the potential benefit of IFNAR blockade in patients with predominantly inflammatory NPSLE manifestations. Further evidence is needed to substantiate this hypothesis.

All eight cases successfully treated with anifrolumab were refractory to previous treatments and showed high disease activity in other organs/systems, regardless of NP involvement. Based on these characteristics, all of the NPSLE cases successfully treated with anifrolumab can be categorized as having inflammatory NP events, according to Zirkzee et al. (11). The NPSLE manifestations successfully treated with anifrolumab included CIDP, ACS, seizures, mood disorders, aseptic meningitis, and psychosis, which are usually considered driven by an inflammatory pathway (7, 11). The co-occurring disease manifestations and the need for minimizing PDN dose may have influenced the decision to use anifrolumab as a treatment option. Interestingly, anifrolumab was also effective in our patient with SLE and APS suffering from partial seizures, which were deemed secondary to brain stroke, successfully treated with oral anticoagulant and anti-epileptic drugs under the assumption of ischemic damage mediated by aPL. However, many years later, the new onset of generalized seizures, worsening during systemic SLE flares with high disease activity, and the acquired refractoriness to anti-epileptic drugs support attributing this NP event to an inflammatory pathogenic pathway (11). Moreover, the aPL and especially the anti-β2GP1 are brain-reactive antibodies that can trigger neuroinflammation and were found to be associated with inflammatory NPSLE events such as seizures, cognitive impairment, psychosis, and depression (38, 39). Regarding anifrolumab efficacy in NP manifestations, it is still highly possible that in some cases the background therapy with GCs or other drugs played a role in leading NPSLE to remission. However, 7 out of 8 retrieved cases (patients 2–8 in **Table 1**) were refractory to multiple immunosuppressants and GC treatments, and clinical response was achieved only by adding anifrolumab to the background treatment (35–37). In 4 out of 8 cases (patients 1, 2, 4, and 7), assessing anifrolumab’s efficacy was challenging because patients were also treated with high-dose GCs, intravenous cyclophosphamide, or immunoglobulins (34–37). The observed rapid improvement in NP symptoms, together with favorable effects on systemic disease activity, clinical remission, and flare prevention, may indicate a therapeutic benefit of anifrolumab as an adjunctive option in the treatment strategy for inflammatory NPSLE. These findings should be taken with caution, as they are based on a limited number of case reports. Nevertheless, they provide, albeit preliminary, support for targeting the type I interferon pathway in inflammatory NPSLE. Moreover, anifrolumab appeared to be well tolerated in the reported cases, apart from the risk of viral infections, consistent with safety data from clinical trials, *post-hoc* analyses, and real-world evidence (40, 41).

This study has some limitations. First, we based our conclusions on successful case reports from the literature, which may be subject to publication bias, as unsuccessful results are less likely to be published. Second, specific data on the effects of blocking IFN-α have been obtained from murine SLE models and from computational analyses. While these models help study the broader NPSLE population, they pose challenges for investigating specific manifestations of NPSLE. Finally, the small number of cases retrieved from the SLR and the high variability in NPSLE manifestations reported suggest that further evidence is needed on the potential effects of anti-IFNAR in NPSLE to yield more consistent data and draw more definitive conclusions about the use of anifrolumab in such severe manifestations.

In conclusion, these preliminary observations suggest a potential role for anifrolumab in the treatment of NPSLE. However, the current evidence remains insufficient to establish efficacy, and controlled studies are needed before anifrolumab can be considered a formal therapeutic option.

## Data Availability

The original contributions presented in the study are included in the article/supplementary material, further inquiries can be directed to the corresponding author/s.
